# Elucidation of Non-Intentionally Added Substances from Plant Fiber/Plastic Composites by UPLC-QTOF/MS

**DOI:** 10.3390/foods12030678

**Published:** 2023-02-03

**Authors:** Hong Zhang, Qi-Zhi Su, Gui-Qin Shang, Yun-Xuan Weng, Lei Zhu

**Affiliations:** 1 School of Light Industry, Beijing Technology and Business University, Beijing 100048, China; 2 China National Center for Food Safety Risk Assessment, Beijing 100022, China; 3 National Reference Laboratory for Food Contact Material (Guangdong), Guangzhou Customs Technology Center, Guangzhou 510623, China; 4 Nanjing Customs Testing Center for Dangerous Goods and Packaging, Changzhou 213000, China; 5 College of Chemistry and Materials Engineering, Beijing Technology and Business University, Beijing 100048, China; 6 Beijing Key Laboratory of Plastic Hygiene and Safety Quality Evaluation Technology, Beijing 100048, China

**Keywords:** plant fiber, melamine formaldehyde resin, PLA, oligomer, derivative, spectral similarity

## Abstract

Plant fiber/plastic composites (PPCs) have been widely used in food contact materials (FCMs) for many benefits, such as their claimed better environmental footprint compared to conventional plastics. However, their safety is still not fully understood and must be comprehensively evaluated. Non-volatiles extracted from six PPCs with different plant fibers and polymer matrices were characterized by employing ultra-high-performance liquid chromatography coupled to quadrupole time-of-flight mass spectrometry in combination with various spectral libraries and manual elucidation, taking into account spectral similarity and characteristic product ions. A total of 115 compounds were tentatively identified, 50 of which were oligomers or their derivatives from the sample with polylactic acid (PLA) and polybutylene adipate terephthalate (PBAT) as the polymer matrix, and some of them were Cramer rules class III substances based on the threshold of toxicological concern (TTC). Seven reaction products between PLA and PBAT monomers, as well as four derivatives of melamine, were elucidated and well detailed for the first time. In addition, bisphenol S was detected in all samples even though its origin remains to be further explored. Isoprothiolane, as an insecticide and fungicide used to control a range of rice pests, was identified in the sample with rice husk as fillers, experimentally confirming the presence of agrochemicals in samples containing plant fibers.

## 1. Introduction

Plant fiber/plastic composites (PPCs), which refer to materials comprising polymer matrices and plant-derived fibers as reinforcement/filler(s), have been employed in a wide variety of applications, such as construction, automobile, food-contact materials (FCMs), and so on, owing to their low-cost nature, being lightweight, and ease of processing [1]. As renewable and sustainable resources, plant fibers, for example, flax, jute, sisal, cotton, bamboo, and rice husk, are abundant, cheap, biodegradable, and easily available. Their utilization in the composites is deemed environmentally friendly with respect to sustainability, carbon emission, and waste management [2,3,4]. Depending on the types of plastics used, PPCs can be either biodegradable or non-biodegradable, with the former being more sustainable and the latter being more durable [5], but both types have been widely applied to the manufacture of diverse FCMs [1] and marketed as sustainable alternatives to conventional plastics [6]. 

Despite the aforementioned advantages, however, PPCs should not endanger consumer health by transferring their constituents into the contacting food like other FCMs [7]. Nature-derived does not necessarily mean safe, as plant fibers may contain many bioactive components, some of which could be allergens or even toxins [8,9]. In addition, pesticides applied during plant cultivation or antimildew agents used during storage could remain in the plant fibers and, consequently, could be present in PPCs [1]. Additionally, additives used in plastic production, such as antioxidants in polypropylene (PP) [10,11] and plasticizers in polylactic acid (PLA) [12], monomers or oligomers of some plastics, as well as impurities, degradation, or reaction products of raw materials, could exist in PPCs [13,14,15]. Almost all plant fibers have a hydrophilic nature as a result of rich hydroxyl (OH) and carboxylic acid (COOH) groups in heteropolysaccharides. Hence, compatibilizers or coupling agents are generally required for the manufacture of PPCs to achieve higher compatibility between the polymer matrix and the plant fibers, and to improve the properties of the PPCs [4]. Any substance present in PPCs could migrate out into the contacting media and consequently pose potential risk to the consumer. 

The potential risk of PPCs has been addressed in recent years. Cellulose- and starch-containing FCMs were reported to have more chemical features and stronger in vitro toxicity [6]. According to a report by [16], 19.8% of the investigated melamine formaldehyde resins (MF) had melamine migration over the specific migration limit (SML) in the third migrate, samples with natural fillers showed higher release, and 5.4% of them exceeded group SML for formaldehyde. Coincidentally, the excessive migration of formaldehyde and melamine from plant-fiber-filled MF from various countries were reported by the Rapid Alert System for Food and Feed as well. Most of the studies in the literature have focused on the determination of monomers [17], oligomers [18], and sometimes the additives of the plastics [19], while reports on non-intentionally added substances (NIAS) [20] such as side reaction products during polymerization, pesticide residues, and bioactive compounds in plant fibers, are rare to the best of the authors’ knowledge. All of these compounds could be responsible for the risk, if any, of the materials, and the characterization of them is the very first step for future safety evaluation.

To comprehensively investigate these substances, non-target screening (NTS), which normally requires high-resolution mass spectrometry (HRMS), is by far the best practice and most used technology. However, NTS is still challenging, particularly for non-volatile substances, which relies heavily on spectral libraries and the experience of the analyst regarding mass spectrometry and the materials under study [13,21,22]. There are increasing numbers of spectral libraries available to the public. For instance, the GNPS library contains plenty of mass spectra of components from nature products and many others, such as pesticides (https://ccms-ucsd.github.io/GNPSDocumentation/gnpslibraries/, accessed on 3 April 2022), which can be of great help to characterize substances from plant fibers. Nonetheless, they have not been well leveraged for the analysis of PPCs. 

The objective of this article is to comprehensively characterize non-volatile substances from six plant fiber/plastic composites by employing ultra-high-pressure liquid chromatography coupled to quadrupole time-of-flight mass spectrometry (UPLC-QTOF/MS). Compounds were firstly extracted with three solvents: ethanol, isooctane, and 4% acetic acid. The extracts were then analyzed by UPLC-QTOF/MS to obtain representative mass spectra of each compound for further identification. We focused on the characterization of non-volatile substances, firstly by leveraging various publicly available libraries (NIST 20, as well as home-made MS/MS spectral libraries) and secondly by manual elucidation, taking advantage of spectral similarity and HRMS for the measurement of exact mass, isotope ratio, and fragment ions of detected signals. The elucidation of PLA and polybutylene adipate terephthalate (PBAT) oligomers and their derivatives, as well as melamine derivates, have been detailed addressing their characteristic product ions, which can be used for the discrimination of some isomers, and spectral similarity. 

## 2. Materials and Methods

### 2.1. Reagents and Samples

Acetic acid (analytical reagent), ethanol, and HPLC-grade isooctane were purchased from Macklin (Shanghai, China). LC-MS-grade formic acid and HPLC-grade methanol were purchased from Thermo Fisher Scientific (Waltham, MA, USA). Six plant fiber/plastic composites used for food contact purposes, in either bowl or cup forms, were purchased from the internet as well as in local markets in Beijing, China. These samples were manufactured by various companies and details about them are listed in Table 1. The plant fibers were from bamboo or rice husk, while the plastics were either PP, MF, or PLA. 

### 2.2. Compound Extraction

All samples were firstly milled into powders under a cryogenic condition and mixed well to obtain homogeneous and representative specimens. Each sample was then weighed (1 ± 0.01 g) into a glass vial with stopper, followed by adding 10 mL of solvent. Solvents used were ethanol, isooctane, and 4% acetic acid, but the last was only applied to MF-based samples (1# and 2#). Subsequently, the samples were sealed well and subjected to extraction. For the former 2 solvents, the extraction was carried out at 40 °C for 1 h, and 1 mL of extract filtered by a 0.22 µm nylon filter was finally subjected to injection. Isooctane was evaporated until dryness and the residue was then re-dissolved with methanol prior to analysis. The 4% acetic acid extraction was conducted at 60 °C for 2 h under an ultrasonic bath and the extract was transferred to a 50 mL volumetric flask. Next, the sample was rinsed with 40 mL of 4% acetic acid. The washing media was subsequently merged with the extract and the volume was brought to 50 mL using fresh 4% acetic acid. One milliliter of the solution was prepared for later injection. Samples were prepared in duplicates and procedural blanks were simultaneously prepared.

### 2.3. Analysis of Non-Volatile Substances by UPLC-QTOF/MS

Non-volatile substances were analyzed by a UPLC-QTOF/MS (1290-6546; Agilent, CA, US) with a resolution of 34000 FWHM, which was equipped with a ZORBAX SB-C18 column (2.1 × 100 mm with 1.8 µm particle size; Agilent J&W Scientific, CA, USA). Injection volume was 3 μL. Separation was conducted at 40 °C with a flow rate of 0.3 mL/min. Mobile phases were water (phase A) modified with 0.1 % formic acid and methanol (phase B). Elution gradients were as follows (35 min in total): kept 5% B for 1.5 min and increased to 40% B in 13.5 min; then further climbed to 98% B in 10 min and maintained for 7 min; finally dropped back to 5% B and remained constant for 2.9 min. Electrospray ionization (ESI) operating in both positive and negative modes was employed. The ion source was operated at 320 °C with nitrogen used as drying (35 psi) and nebulizing gas (8 L/min). Capillary, fragmentor, skimmer, and OCT 1 RF Vpp voltages were 3.5 kV, 140 V, 65 V, and 750 V, respectively. Data-dependent acquisition (DDA) was applied to both MS^1^ and MS^2^ scanned from 50–1000 m/z at a rate of 4 spectra/s.

### 2.4. Data Processing and Compound Identification

The UPLC-QTOF/MS data were processed by MS-DIAL (version 4.90) (Tsugawa et al., 2015). MS^1^ and MS^2^ tolerances were set as 0.01 and 0.025 Da, respectively, and minimum peak height was 1000. The adducts [M − H]^−^, [M + FA − H]^−^, [M + Hac − H]^−^, [2M − H]^−^, [2M + FA − H]^−^, and [2M + Hac − H]^−^ were selected in negative mode, while [M + H]^+^, [M + NH4]^+^, [M + Na]^+^, [M + K]^+^, [2M + H]^+^, [2M + NH4]^+^, [2M + Na]^+^, and [2M + K]^+^ were in positive mode. For peak alignment, retention time and MS1 tolerances were 0.05 min and 0.015 Da, respectively. Moreover, only peaks that had sample max/blank average fold change higher than 5 were kept. Characterization of non-volatile substances was firstly carried out by comparing acquired spectra against MS/MS libraries leveraging both commercial, i.e., NIST 20 and home-built MS/MS libraries. Publicly accessible libraries from MoNA (https://mona.fiehnlab.ucdavis.edu/, accessed on 3 April 2022), and GNPS [23] were integrated as well. For identification, MS^1^ and MS^2^ mass tolerance were 0.01 and 0.05 Da, respectively, and the identification score cut-off was 75. Additionally, melamine derivatives, oligomers, and their derivatives were manually elucidated, taking exact mass, isotope ratio, characteristic fragment ions, and similarity of the spectrum to that of previously identified compounds into consideration. The calculation of molecular formulas was carried out by MS-FINDER (version 3.52) considering C, H, O, N, and S elements, which were thought to be the most common elements in food contact materials unless other specific elements were suspected [13]. Cramer rules classification was carried out by Toxtree 3.1.0.1851 [24].

## 3. Results and Discussions

### 3.1. Identification by Matching against MS/MS Libraries

**Figure 1:**
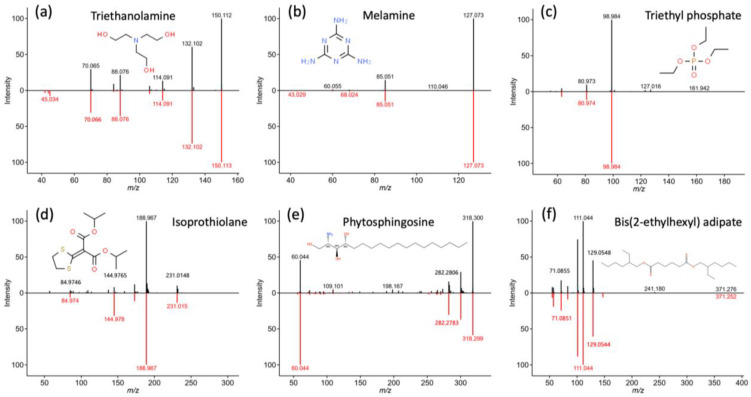
Mirror plots of the acquired MS/MS spectra (black) of six representative compounds against reference spectra (red) in compiled libraries, as detailed in Section 2.4. (**a**) Triethanolamine; (**b**) Melamine; (**c**) Triethyl phosphate; (**d**) Isoprothiolane; (**e**) Phytosphingosine; (**f**) Bis(2-ethylhexyl) adipate.

As demonstrated in Table 2, a total of 115 non-volatile compounds were tentatively identified in these samples, considering both positive and negative modes. In addition, their specific migration limits (SMLs) or Cramer rules classifications are given in the table as well. Thirty-two compounds were characterized by comparing against the compiled spectral libraries. It is reasonable to discover acetyl tributyl citrate (ATBC) in PLA-based composites as it is widely used as a plasticizer in PLA [12]. However, as another citrate ester, tributyl aconitate has been reported to be a hydrolysis product of ATBC and therefore a NIAS [25]. As examples, Figure 1 displays the mirror plots of acquired spectra (black) of six representative compounds against reference spectra (red). While ATBC only presented in the PLA-based sample, another plasticizer, namely bis(2-ethylhexyl) adipate, was identified in all samples, which implies the commonality of plasticizers in PPCs for compounding purposes [1]. It was reported that surfactants such as perfluorooctane sulfonate (PFOS) and perfluorooctanoic acid (PFOA) can be used in the production of plant-fiber-based materials [26]. They were not identified herein, though another commonly used surfactant, namely triethanolamine, was characterized in all samples. It must be mentioned that triethanolamine has a low SML, i.e., 0.05 mg/kg in the EU 10/2011 regulations [27], suggesting a relatively high safety concern. In line with a previous study [20], melamine, a primary building block of the MF, was detected. Moreover, triethyl phosphate, which can be used as a plasticizer for resins or as a raw material to prepare insecticides, was identified in the sample with PLA matrix (#6). Consistent with a preliminary study [1] that pesticide residues can be present in plant fibers, isoprothiolane, which is employed as an insecticide and fungicide to control a range of rice pests, was identified in sample #5 with rice husk as fillers. This finding experimentally confirmed the presence of pesticides in PPCs. Aside from these compounds, many plant-related substances were qualified by matching to libraries, e.g., phytosphinosine, syrinaldehyde, and syringaresinol diglucoside, amongst others. Many of these substances, as well as triethyl phosphate and isoprothiolane, have no SML, though they were classified as Cramer rules III, suggesting potential risks.

Unexpectedly, bisphenol S (BPS), which might have similar or less endocrine disruption toxicity compared to bisphenol A (BPA), and has a SML of 0.05 mg/kg [28], was identified and confirmed with reference standard in all samples investigated. BPS is commonly used in polymerization reactions and epoxy glues, as an anti-corrosive reagent, or as a claimed safer alternative to BPA in thermal paper production, acting as a color developer [29,30]. It has previously been detected in paper products [30,31], canned foodstuffs [29], or even in plants [28]. Nevertheless, this is the first time its occurrence has been reported PPCs to our knowledge, which is unexpected, but its origin remains to be explored.

### 3.2. Elucidation of Melamine Derivatives

In accordance with a previous study by Osorio [20], some melamine derivatives besides melamine were observed in the MF-based samples (#1 and #2). Nonetheless, no characteristic product ions or representative spectra of those derivatives nor elucidation details were given in that research. In addition, most of the melamine derivatives reported were not observed in this study even by directly searching their precursor ions in MS-DIAL, which suggests that depending on the sample, and possibly the production process, melamine derivatives in MF can vary. Instead, new melamine derivatives were observed and identified for the first time in this article. Elucidation details are shown below, which can be helpful for understanding the characteristic ions and forming mechanisms of these compounds.

**Figure 2:**
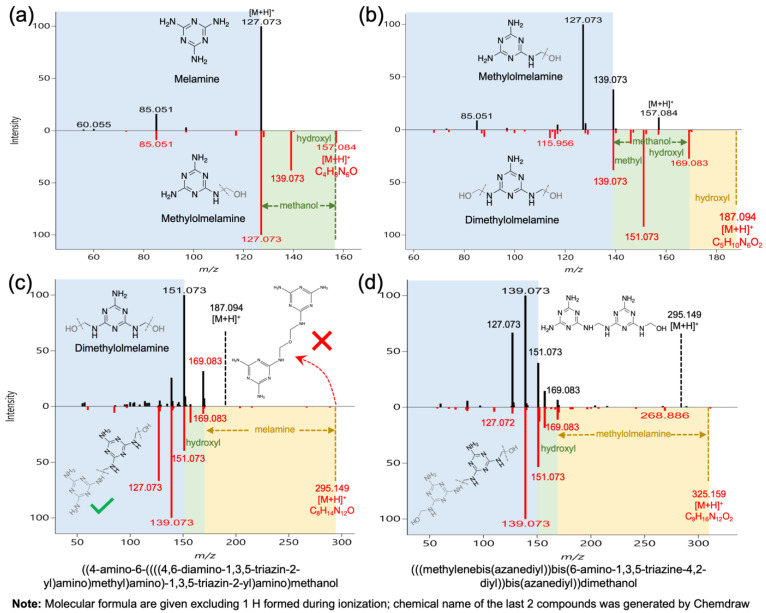
Elucidation of four melamine derivatives based on their similarity in MS/MS spectra. (**a**) Methylolmelamine; (**b**) Dimethylolmelamine; (**c**) ((4-amino-6-((((4,6-diamino-1,3,5-triazin-2-yl)amino)methyl)amino)-1,3,5-triazin-2-yl)amino)methanol; (**d**) (((methylenebis(azanediyl))bis-(6-amino-1,3,5-triazine-4,2-diyl))bis(azanediyl))dimethanol.

In general, these derivatives were only detected in samples #1 and #2 as expected, and they all had [M + H]^+^ as the predominant adduct because the adduct-forming ability of these compounds was strongly suppressed by the competition from protonation [32]. Similarly, methylolmelamine was assigned to the precursor ion 157.084 m/z as depicted in Figure 2a, which had a formular C_4_H_8_N_6_O and quite a similar MS/MS spectrum compared to melamine. Apart from the two common product ions, i.e., 85.051 and 127.073 (formed by losing one methanol), 139.073 was formed by losing a hydroxyl group. With this in mind, methylolmelamine was the most probable structure for this peak. Regarding the peak 187.094 m/z with a formular C_4_H_8_N_6_O (Figure 2b), 127.073 was not observed, though 139.073 was significant, suggesting a similar structure to methylolmelamine. Moreover, the difference between 139.073 and 151.073 was 12, which corresponds to another methyl group considering potential hydrogen rearrangement during fragmentation [33]. Theoretically, this methyl group can be linked either to the previous methyl group or another amino group. However, the presence of 169.083 supported the latter case, as it can be explained by dimethylolmelamine with a loss of a hydroxyl group, while the former case cannot. As such, dimethylolmelamine was assigned to the peak 187.094 m/z. It should be noted that these two methylol groups can be connected to either a single amino group or two separated amino groups. However, they are not differentiable by MS/MS spectra alone, and the latter was selected herein. These compounds are the result of the melamine and formaldehyde reaction which is the basis of MF production [34]. In theory, there could be up to six methylol groups connected to the melamine (two methylol to each amino group); however, no more were found in these samples after carefully checking their theoretical precursor ions in MS-DIAL.

With respect to the peak 295.149 m/z with a formular C_8_H_14_N_12_O (Figure 2c), it could be misidentified as a substructure of MF (refer to the structure shown in top-right corner) characterized by a dimethylene–ether bridge. It could be a degradation product, side product because of insufficient polymerization, or reaction product of two methylolmelamines [34]. Nevertheless, as discussed above, the product ion 151.073, which corresponds to a melamine with two methyl groups linked to two separate amino groups, cannot be explained by this structure. Hence, another candidate, named ((4-amino-6-((((4,6-diamino-1,3,5-triazin-2-yl)amino)methyl)amino)-1,3,5-triazin-2-yl)amino)methanol, was proposed as demonstrated in the bottom-left corner, which can be a reaction product of melamine and dimethylolmelamine characterized by a methylene bridge [34]. In this way, all major product ions were explainable, and therefore it was considered as the most probable candidate. Following a same pattern, the peak 325.159 m/z with a formular C_9_H_16_N_12_O_2_ was identified as (((methylenebis(azanediyl))bis- (6-amino-1,3,5-triazine-4,2-diyl))bis(azanediyl))dimethanol. This compound differs from the last compound by a methylol group, as depicted in Figure 2d, and could be a reaction product between methylolmelamine and dimethylolmelamine. Both compounds contained the product ions 127.073, 139.073, 151.073, and 169.083 m/z with a slight difference in intensity. As far as we know, this is the first time that these two melamine derivatives in MF-based samples have been reported. It is worth mentioning that all of the last three melamine derivatives did not have precursor ions in their MS/MS spectra, suggesting that they might be prone to fragmentation, while the first derivative, i.e., methylolmelamine, had quite low precursor ion intensity in its MS/MS spectrum. Another observation of potential interest was that derivatives with two methylol groups had quite low or even invisible 127.073 m/z product ion, while the ones with one methylol group had much higher 127.073 m/z, but the underlying mechanism remains to be investigated. Furthermore, all four of these derivatives were grouped as Cramer rules III substances, and warrant further migration studies and risk assessment to determine their actual safety risks.

### 3.3. Elucidation of PLA Oligomers and Their Derivatives

As expected, a large number of PLA and their derivatives, both linear and cyclic, were tentatively identified in the PLA-based sample (#6), as shown in Table 2 [35]. To be more intuitive, they were named after the type of the molecule, either cyclic or linear, plus the number of each monomer. For example, Linear 5LA represents five repeating lactic acid (LA) units in linear form. All these oligomers and their derivatives were observed in positive mode, except for Linear 2A, which was only ionized in negative mode. Additionally, most of the linear oligomers were detected in both positive and negative modes, whilst cyclic ones were only detected in positive mode. These phenomena could be attributed to the high acidity of linear oligomers with one hydroxyl and one carboxylic acid group at each end, which are favorable for deprotonation [36]. For oligomers with the same number of LA repeating units, cyclic oligomers had smaller molecular weight (one H_2_O less), though they had significantly longer retention time; e.g., Linear 5LA had 16.946 min while Cyclic 5LA had 18.812 min, probably because linear oligomers had higher polarity and therefore moved faster in reverse-phase chromatography. Additionally, cyclic oligomers generally have bigger steric hindrance, making them move slower.

**Figure 3:**
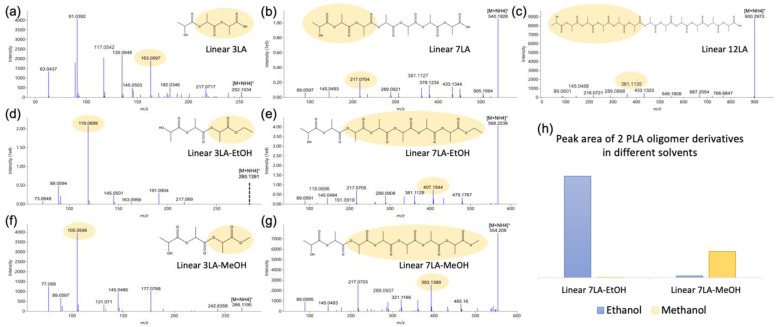
Spectra of linear PLA oligomers and their esterification products with ethanol and methanol. (**a**) Linear 3LA; (**b**) Linear 7LA; (**c**) Linear 12LA; (**d**) Linear 3LA-EtOH; (**e**) Linear 7LA-EtOH; (**f**) Linear 3LA-MeOH; (**g**) Linear 7LA-MeOH; (**h**) peak area of Linear 7LA-EtOH and Linear 7LA-MeOH in extracts with ethanol and methanol as solvents.

Figure 3a–c show the MS/MS spectra of PLA oligomers with different degrees of polymerization. Amongst others, 89.0239, 145.0501, 217.0712, 289.0923, and 361.1135 were characteristic in PLA oligomers depending on the number of repeating units. Different from larger oligomers (Figure 3b,c), which had much higher precursors than product ions in the tandem mass spectra, smaller oligomers, such as Linear 3LA (Figure 3a), had additional fragments such as 63.0437, 91.0392, 117.0542, 135.0648, and 163.0597, and much lower intensity of precursor ions, possibly because bigger molecules needed more collision energy to break this down, while this energy was excessive for smaller oligomers and hence were able to fragment product ions further. Similar to a previous study [37], many linear PLA oligomers combined with an ethyl group were observed (Figure 3d,e). Nonetheless, in disagreement with the authors, we deemed it more reasonable to have the ethyl group linked to the carboxylic acid side of the oligomer rather than to the hydroxyl side because these compounds could be the result of esterification products of linear PLA oligomers and ethanol, which was the solvent for compound extraction. Outside of our expectations, esterification products of linear PLA oligomers and methanol were found as well (Figure 3f,g). Interestingly, the esterification products with ethanol were only observed in ethanol extracts, while those with methanol were merely present in methanol extracts, as demonstrated in Figure 3h. Methanol was not employed to extract compounds from samples, though it was applied for compound extraction from isooctane extracts as isooctane is not compatible with UPLC. It is noteworthy that esterification products of PLA oligomers and ethanol were only observed in 95% ethanol migrates as well, and no esterification product of PLA oligomers and methanol was found even by directly searching their precursor ions in the abovementioned study [37]. This result suggests that more NIAS could be present if the sample under investigation contains compounds with hydroxyl groups. Except for those common fragments in linear PLA oligomers, esterification products of linear PLA and ethanol or methanol had their own characteristic product ions; for example, 119.0699 in Linear 3LA-EtOH, 407.1544 in Linear 7LA-EtOH, 105.0546 in Linear 3LA-MeOH, and 393.1389 in Linear 7LA-MeOH, as shown in Figure 3d–g.

### 3.4. Elucidation of PBAT Oligomers and Their Derivatives

Neat PLA is known to have poor impact and tear resistance, and therefore modification is normally required. PBAT, which is a biodegradable aliphatic–aromatic copolyester and has super-tough properties with a very low tensile modulus, is one of the most employed modifiers for improving the mechanical properties of PLA [38]. Adipic acid (AA), 1,4-butanediol (BD), and terephthalic acid (TPA) are the three monomers of PBAT. Not surprisingly, numerous PBAT oligomers were identified in the PLA-based samples (#6) as well. Similar compounds were observed in two PLA-based biopolymers, and the authors considered them as compounds that have phthalic acid (PA) as one of the substructures [37]. However, as far as we are concerned, taking the popularity of PBAT in PLA into consideration, it is more appropriate to consider them as PBAT oligomers, which have TPA rather than PA as a building block even though both can have 149.0239 m/z as a characteristic product ion.

**Figure 4:**
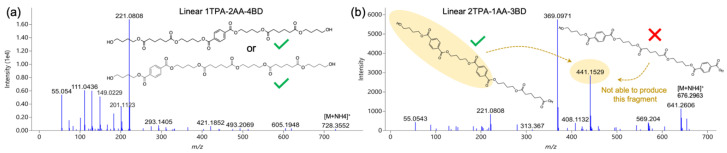
Discrimination of isomers of PBAT oligomers by MS/MS spectrum: (**a**) two isomers of Linear 1TPA-2AA-4BD not discriminated; (**b**) isomers of Linear 2TPA-1AA-3BD differentiated by a characteristic product ion (441.1529).

Unlike PLA oligomers, PBAT oligomers could be more complex as they have three monomers as building blocks, and theoretically could be involved in more combinations. Taking linear form as an example, for a given number of monomers, PLA oligomers are unique, while PBAT could have different isomers differing in the arrangement of monomers, some of which might not be able to be differentiated by MS/MS alone and no precise chemical structure can be given. For example, Linear 1TPA-2AA-4BD can be either Linear BD-AA-BD-TPA-BD-AA-BD or Linear BD-TPA-BD-AA-BD-AA-BD, and both, in theory, may have characteristic product ions, such as 55.054, 111.0436, 129.0549, 149.0229, 183. 1021, 201.1123, and 221.0808 (Figure 4a). Notwithstanding, some isomers are differentiable by MS/MS spectra since they can have their own characteristic product ions that may not be generated by other isomers. For instance, two isomers of Linear 2TPA-1AA-3BD were discerned by a characteristic fragment (441.1529), which can only be produced by the structure shown in the left side of Figure 4b, i.e., Linear BD-TPA-BD-TPA-BD-AA, while the one in the right side might not be able to produce this fragment. Following the same way, many more isomers were assigned to unique structures, while some remained unknown, and only one of them was selected as a representative structure.

**Figure 5:**
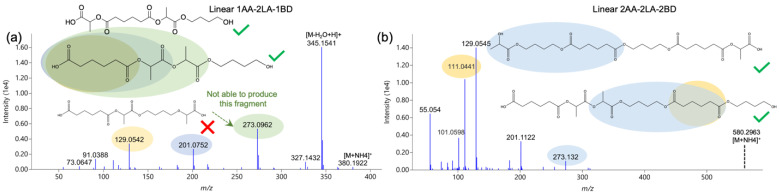
Representative spectra of PBAT oligomer derivatives. (**a**) Linear 1AA-2LA-1BD; (**b**) Linear 2AA-2LA-2BD.

Apart from PLA and PBAT oligomers, combinations of AA, LA, and BD, which could be the result of side-reaction products during PLA-PBAT blending, were also observed and identified. These compounds can be regarded as derivatives of either PLA or PBAT oligomers. To the best of our knowledge, this is the first time they have been reported in PLA-based materials. Figure 5 shows the representative spectra of two of them. Fragment 201.0752 is the combination of 1AA and 1LA, while 273.0962 is the result of bonding 1AA and 2LA together. This is helpful to exclude the candidate shown at the bottom of Figure 5a but not to discern the other two. Linear 2AA-2LA-2BD had product ions 201 and 273 as well, but they were not the same as the previous one if we look at their exact mass (201.1122 and 273.132 but not 201.0752 and 273.0962). Thanks to the power of HRMS, we can observe this discrepancy. However, the two isomers were still indifferentiable. It is interesting that derivatives containing a TPA component were not observed.

As evidenced by the large number of PLA and PBAT oligomers, as well as their derivatives, PLA-based materials are far more complex than other plastics in terms of chemical diversity. Many of the oligomers have Cramer rules class III as showcased in Table 2, and most of them, if not all, have not been toxicologically evaluated by experiments, and hence little is known about their risk, if any.

## 4. Conclusions

Non-volatile substances from six plant fiber/plastic composites (PPCs) were extensively characterized by ultra-high-performance liquid chromatography coupled to quadrupole time-of-flight mass spectrometry. A number of compounds, including plant components, plasticizers, and surfactants used for polymer production were identified, leveraging many tandem mass spectral libraries, which proves the value of these resources for compound identification from PPCs. The observation of isoprothiolane, which is used to control rice pests, experimentally confirmed the possibility of pesticide residues in PPCs. Furthermore, the presence of Bisphenol S in all samples was outside of expectations, and its origin is worth further exploration.

Besides methylolmelamine and dimethylolmelamine, which were expected, two new melamine derivatives were tentatively identified in the melamine–formaldehyde-based PPC in a step-by-step manner, taking the exact mass of the precursor and characteristic product ions, as well as spectral similarity, into consideration. These derivatives could be the reaction products of dimethylolmelamine and melamine/methylolmelamine. Compared to other plastic-based PPCs, PLA-based materials showed many more compounds detected and identified; mostly oligomers and their derivatives. Linear PLA oligomers were found to be able to react with the solvent used for extraction, in this case methanol and ethanol, suggesting that they could react with other compounds with hydroxyl groups if present. Reaction products of PLA monomer (LA) and PBAT monomers (adipic acid and 1,4-butanediol) were also identified for the first time, which further complicate the chemical complexity of PLA-based materials. Many of the PLA or PBAT oligomers and their derivatives, in theory, can have various isomers varying in the arrangement of their monomers. Some of them were discerned by characteristic product ions, while others remained indiscriminable. The use of ion mobility and collision cross section (CCS) could be of help in this regard. It should be pointed out that ethanol was one of the solvents for the extraction, which could lead to alcoholysis of PLA and PBAT and therefore overestimate the extraction of PLA and PBAT oligomers and their derivatives.

In summary, this work is the very first step to understanding the potential hazards posed by PPCs. The use of various spectral libraries, as well as the detailed elucidation given, can be of great help for future investigations with respect to compound identification. Nevertheless, there is still a lot of work to be done towards a comprehensive understanding of the safety of PPCs; for example, the quantification and risk assessment of these oligomers, considering that most of them have no reference standards commercially available.

## Figures and Tables

**Table 1:** Detailed information about the plant fiber/plastic composite samples.

Number	Plant Fiber	Plastic	Type	Weight (g)	Area (dm^2^)
1#	Bamboo	MF	Bowl	86	1.9
2#	Bamboo	MF	Bowl	212	3.2
3#	Bamboo	PP	Cup	125	3.4
4#	Rice husk	PP	Bowl	238	3.4
5#	Rice husk	PP	Bowl	128	1.9
6#	Rice husk	PLA	Cup	78	1.7

Note: # is a symbol of sample code; MF, PP, and PLA in the Plastic column are abbreviations of melamine formaldehyde resin, polypropylene, and polylactic acid, respectively; Area represents the internal surface area of the containers.

**Table 2:** Non-volatile substances tentatively identified in plant fiber/plastic composites.

Nº	RT (min)	Precursor Ion	Adduct	Name	CAS	Formula	SML or CR	Score	Sample
1	2.12	150.1127	[M + H]^+^	Triethanolamine	102-71-6	C_6_H_15_NO_3_	0.05	97	E; #1, #2, #3, #4, #5, #6
2	2.33	127.0732	[M + H]^+^	Melamine	108-78-1	C_3_H_6_N_6_	2.5	93	E, H; #1, #2
3	2.36	365.1048	[M + Na]^+^	Sucrose	57-50-1	C_12_H_22_O_11_	III	86	E; #1, #2, #3, #4, #5, #6
4 *	2.62	157.0835	[M + H]^+^	Methylolmelamine	937-35-9	C_4_H_8_N_6_O	III	-	H; #1, #2
5	3.02	144.0653	[M + H − H2O]^+^	N-Methyl-L-glutamic acid	35989-16-3	C_6_H_11_NO_4_	III	81	E; #5
6 *	3.29	187.0937	[M + H]^+^	Dimethylol melamine	5001-80-9	C_5_H_10_N_6_O_2_	III	-	H; #1, #2
7 *	4.45	295.1487	[M + H]^+^	((4-amino-6-((((4,6-diamino-1,3,5-triazin-2-yl)amino)methyl)amino)-1,3,5-triazin-2-yl)amino)methanol	-	C_8_H_14_N_12_O	III	-	H; #1, #2
8 *	5.68	325.1593	[M + H]^+^	(((methylenebis(azanediyl))bis(6-amino-1,3,5-triazine-4,2-diyl))bis(azanediyl))dimethanol	-	C_9_H_16_N_12_O_2_	III	-	H; #1, #2
9º	8.12	161.0463	[M − H]^−^	Linear 2LA	-	C_6_H_10_O_5_	I	-	E; #6
10	8.63	250.1439	[M + H]^+^	4-Coumaroylcholine	-	C_14_H_20_NO_3_	III	87	E; #1, #2, #3, #6
11	8.84	171.0124	[M − H]^−^	p-Toluenesulfonic acid	104-15-4	C_7_H_8_O_3_S	I	81	E, M; #6
12	8.90	359.0979	[M − H]^−^	1-O-(3-Hydroxy-4,5-dimethoxybenzoyl)hexopyranose	-	C_15_H_20_O_10_	III	76	H; #1
13	9.00	145.0510	[M − H]^−^	adipic acid	124-04-9	C_6_H_10_O_4_	I	94	E; #6
14	9.46	137.0245	[M − H]^−^	2,5-Dihydroxybenzaldehyde	1194-98-5	C_7_H_6_O_3_	I	89	E; #4, #6
15	9.91	327.1081	[M − H]^−^	3-(4-Hydroxyphenyl)-3-oxopropyl.beta.-D-glucopyranoside	-	C_15_H_20_O_8_	III	81	H; #1
16	10.27	137.0247	[M − H]^−^	4-Hydroxybenzoic acid	99-96-7	C_7_H_6_O_3_	I	88	E; #2, #3, #4, #6
17	10.34	165.0556	[M − H]^−^	3,4’-Dihydroxypropiophenone	53170-93-7	C_9_H_10_O_3_	I	78	E; #1, #2, #3, #4, #5, #6
18	10.35	367.1028	[M − H]^−^	5-O-Feruloylquinic acid	62929-69-5	C_17_H_20_O_9_	II	78	E; #2, #3
19	10.48	496.2020	[M + NH4]^+^	Kelampayoside A	87562-76-3	C_20_H_30_O_13_	III	84	E; #2
20	11.34	121.0296	[M − H]^−^	4-Hydroxybenzaldehyde	123-08-0	C_7_H_6_O_2_	I	89	E, H; #1, #2, #3, #4, #5, #6
21º	12.06	252.1078	[M + NH4]^+^	Linear 3LA	-	C_9_H_14_O_7_	I	-	E; #6
22	12.33	367.1026	[M − H]^−^	1,3,5-Trihydroxy-4-(((2E)-3-(3-hydroxy-4-methoxyphenyl)prop-2-enoyl)oxy)cyclohexanecarboxylic acid	-	C_16_H_20_O_10_	III	77	E; #2, #3, #6
23	12.65	183.0651	[M + H]^+^	Syringaldehyde	134-96-3	C_9_H_10_O_4_	I	87	E, M; #1, #2, #3, #4, #5, #6
24º	12.76	252.1442	[M + NH4]^+^	Linear 2LA-1BD	-	C_10_H_18_O_6_	I	-	E; #6
25	12.76	371.0971	[M − H]^−^	3-(Benzoyloxy)-2-hydroxypropyl.beta.-D-glucopyranosiduronic acid	-	C_16_H_20_O_10_	III	77	E; #2, #3, #6
26	12.87	760.3018	[M + NH4]^+^	Syringaresinol diglucoside	96038-87-8	C_34_H_46_O_18_	III	82	H; #1, #2
27	12.89	787.2673	[M + FA − H]^−^	Eleutheroside E	39432-56-9	C_34_H_46_O_18_	III	75	H; #1
28	13.24	163.0405	[M − H]^−^	trans-4-Coumaric acid	501-98-4	C_9_H_8_O_3_	I	94	E; #1, #2, #3, #4, #5, #6
29º	13.64	236.1488	[M + NH4]^+^	Linear 1AA-1BD	-	C_10_H_18_O_5_	I	-	E; #6
30	13.86	147.0456	[M − H − H2O]^−^	3-(2-Hydroxyphenyl)propionic acid	495-78-3	C_9_H_10_O_3_	I	90	E; #1, #2, #3, #4, #5, #6
31	13.99	251.0372	[M + H]^+^	Bisphenol S	80-09-1	C_12_H_10_O_4_S	0.05	89	E, H; #1, #2, #3, #4, #5, #6
32	14.19	207.0667	[M − H]^−^	trans-3,5-Dimethoxy-4-hydroxycinnamaldehyde	4206-58-0	C_11_H_12_O_4_	I	84	E; #1, #2, #3, #4, #5, #6
33	14.45	598.2492	[M + NH4]^+^	(-)-Syringaresinol-4-O-.beta.-D-glucopyranoside	7374-79-0	C_28_H_36_O_13_	III	79	E; #1, #2, #3, #6
34º	14.73	266.1231	[M + NH4]^+^	Linear 3LA-MeOH	-	C_10_H_16_O_7_	I	-	M; #6
35º	14.88	324.1297	[M + NH4]^+^	Linear 4LA	-	C_12_H_18_O_9_	I	-	E; #6
36	15.88	183.0780	[M + H]^+^	Triethyl phosphate	78-40-0	C_6_H_15_O_4_P	III	90	E; #6
37º	15.89	291.1438	[M + H]^+^	Linear 1AA-1LA-1BD	-	C_13_H_22_O_7_	I	-	E; #6
38º	16.28	291.1805	[M + H]^+^	Linear 1AA-2BD	-	C_14_H_26_O_6_	I	-	E; #6
39º	16.68	280.1391	[M + NH4]^+^	Linear 3LA-EtOH	-	C_11_H_18_O_7_	I	-	E, M; #6
40	16.84	441.2018	[M + H]^+^	Diferuloyl putrescine	42369-86-8	C_24_H_28_N_2_O_6_	III	83	E; #1, #2, #3, #6
41º	16.95	396.1513	[M + NH4]^+^	Linear 5LA	-	C_15_H_22_O_11_	I	-	E; #6
42º	16.97	338.1443	[M + NH4]^+^	Linear 4LA-MeOH	-	C_13_H_20_O_9_	I	-	M; #6
43º	17.50	380.1917	[M + NH4]^+^	Linear 1AA-2LA-1BD	-	C_16_H_26_O_9_	I	-	E; #6
44º	17.81	380.2279	[M + NH4]^+^	Linear 1AA-1LA-2BD	-	C_17_H_30_O_8_	I	-	E; #6
45º	18.13	364.1971	[M + NH4]^+^	Linear 2AA-1BD	-	C_16_H_26_O_8_	I	-	E; #6
46º	18.39	468.1723	[M + NH4]^+^	Linear 6LA	-	C_18_H_26_O_13_	III	-	E; #6
47º	18.52	352.1606	[M + NH4]^+^	Linear 4LA-EtOH	-	C_14_H_22_O_9_	I	-	E, M; #6
48º	18.60	410.1658	[M + NH4]^+^	Linear 5LA-MeOH	-	C_16_H_24_O_11_	III	-	M; #6
49º	18.81	378.1398	[M + NH4]^+^	Cyclic 5LA	-	C_15_H_20_O_10_	I	-	E; #6
50º	18.96	452.2493	[M + NH4]^+^	Linear 1AA-2LA-2BD	-	C_20_H_34_O_10_	I	-	E; #6
51º	19.45	436.2546	[M + NH4]^+^	Linear 2AA-2BD	-	C_20_H_34_O_9_	I	-	E; #6
52º	19.54	540.1935	[M + NH4]^+^	Linear 7LA	-	C_21_H_30_O_15_	III	-	E; #6
53º	19.80	482.1865	[M + NH4]^+^	Linear 6LA-MeOH	-	C_19_H_28_O_13_	III	-	M; #6
54º	19.86	424.1816	[M + NH4]^+^	Linear 5LA-EtOH	-	C_17_H_26_O_11_	III	-	E; #6
55º	20.09	384.1652	[M + NH4]^+^	Linear 1TPA-1AA-1BD	-	C_18_H_22_O_8_	I	-	E; #6
56º	20.24	450.1609	[M + NH4]^+^	Cyclic 6LA	-	C_18_H_24_O_12_	I	-	E, M; #6
57º	20.45	612.2145	[M + NH4]^+^	Linear 8LA	-	C_24_H_34_O_17_	III	-	E; #6
58º	20.49	508.3116	[M + NH4]^+^	Linear 2AA-3BD	-	C_24_H_42_O_10_	I	-	E; #6
59º	20.74	554.2076	[M + NH4]^+^	Linear 7LA-MeOH	-	C_22_H_32_O_15_	III	-	M; #6
60º	20.84	496.2035	[M + NH4]^+^	Linear 6LA-EtOH	-	C_20_H_30_O_13_	III	-	E; #6
61º	20.97	580.2963	[M + NH4]^+^	Linear 2AA-2LA-2BD	-	C_26_H_42_O_13_	III	-	E; #6
62º	21.13	580.3326	[M + NH4]^+^	Linear 2AA-1LA-3BD	-	C_27_H_46_O_12_	III	-	E; #6
63º	21.18	689.1904	[M + Na]^+^	Linear 9LA	-	C_27_H_38_O_19_	III	-	E; #6
64º	21.22	522.1820	[M + NH4]^+^	Cyclic 7LA	-	C_21_H_28_O_14_	I	-	E, M; #6
65º	21.23	456.2225	[M + NH4]^+^	Linear 1TPA-1AA-2BD	-	C_22_H_30_O_9_	I	-	E; #6
66º	21.30	564.3017	[M + NH4]^+^	Linear 3AA-2BD	-	C_26_H_42_O_12_	I	-	E; #6
67º	21.48	626.2288	[M + NH4]^+^	Linear 8LA-MeOH	-	C_25_H_36_O_17_	III	-	M; #6
68º	21.53	568.2238	[M + NH4]^+^	Linear 7LA-EtOH	-	C_23_H_34_O_15_	III	-	E; #6
69º	21.61	418.2438	[M + NH4]^+^	Cyclic 2AA-2BD	-	C_20_H_32_O_8_	I	-	E, M; #6
70º	21.72	652.3535	[M + NH4]^+^	Linear 2AA-2LA-3BD	-	C_30_H_50_O_14_	III	-	E; #6
71	21.72	291.0716	[M + H]^+^	Isoprothiolane	50512-35-1	C_12_H_18_O_4_S_2_	III	86	E, M; #5
72º	21.75	756.2562	[M + NH4]^+^	Linear 10LA	-	C_30_H_42_O_21_	III	-	E, M; #6
73º	21.80	594.2036	[M + NH4]^+^	Cyclic 8LA	-	C_24_H_32_O_16_	I	-	E; #6
74º	21.94	636.3594	[M + NH4]^+^	Linear 3AA-3BD	-	C_30_H_50_O_13_	III	-	E; #6
75º	22.01	528.2801	[M + H]^+^	Linear 1TPA-1AA-3BD	-	C_26_H_38_O_10_	I	-	E; #6
76º	22.23	640.2454	[M + NH4]^+^	Linear 8LA-EtOH	-	C_26_H_38_O_17_	III	-	E; #6
77º	22.27	828.2771	[M + NH4]^+^	Linear 11LA	-	C_33_H_46_O_23_	III	-	E; #6
78º	22.49	708.4163	[M + NH4]^+^	Linear 3AA-4BD	-	C_34_H_58_O_14_	III	-	E; #6
79º	22.55	666.2253	[M + NH4]^+^	Cyclic 9LA	-	C_27_H_36_O_18_	I	-	E; #6
80º	22.58	770.2703	[M + NH4]^+^	Linear 10LA-MeOH	-	C_31_H_44_O_21_	III	-	M; #6
81º	22.69	900.2977	[M + H]^+^	Linear 12LA	-	C_36_H_50_O_25_	III	-	E; #6
82º	22.73	712.2661	[M + NH4]^+^	Linear 9LA-EtOH	-	C_29_H_42_O_19_	III	-	E; #6
83º	22.75	584.2704	[M + NH4]^+^	Linear 1TPA-2AA-2BD	-	C_28_H_38_O_12_	I	-	E; #6
84º	22.96	764.4058	[M + NH4]^+^	Linear 4AA-3BD	-	C_36_H_58_O_16_	III	-	E; #6
85º	22.99	842.2909	[M + NH4]^+^	Linear 11LA-MeOH	-	C_34_H_48_O_23_	III	-	M; #6
86º	23.04	972.3190	[M + NH4]^+^	Linear 13LA	-	C_39_H_54_O_27_	III	-	E; #6
87º	23.16	784.2859	[M + NH4]^+^	Linear 10LA-EtOH	-	C_32_H_46_O_21_	III	-	E; #6
88º	23.20	738.2455	[M + NH4]^+^	Cyclic 10LA	-	C_30_H_40_O_20_	I	-	E; #6
89º	23.25	656.3275	[M + NH4]^+^	Linear 1TPA-2AA-3BD	-	C_32_H_46_O_13_	I	-	E; #6
90º	23.36	914.3131	[M + NH4]^+^	Linear 12LA-MeOH	-	C_37_H_52_O_25_	III	-	M; #6
91º	23.37	836.4641	[M + NH4]^+^	Linear 4AA-4BD	-	C_40_H_66_O_17_	III	-	E; #6
92º	23.40	438.2122	[M + NH4]^+^	Cyclic 1TPA-1AA-2BD	-	C_22_H_30_O_9_	I	-	E, M; #6
93º	23.51	856.3078	[M + NH4]^+^	Linear 11LA-EtOH	-	C_35_H_50_O_23_	III	-	E; #6
94º	23.54	728.3851	[M + NH4]^+^	Linear 1TPA-2AA-4BD	-	C_36_H_54_O_14_	I	-	E; #6
95º	23.65	810.2664	[M + NH4]^+^	Cyclic 11LA	-	C_33_H_44_O_22_	I	-	E; #6
96º	23.81	928.3292	[M + NH4]^+^	Linear 12LA-EtOH	-	C_38_H_54_O_25_	III	-	E; #6
97º	23.84	618.3482	[M + NH4]^+^	Cyclic 3AA-3BD	-	C_30_H_48_O_12_	I	-	E, M; #6
98º	24.01	784.3750	[M + NH4]^+^	Linear 1TPA-3AA-3BD	-	C_38_H_54_O_16_	I	-	E; #6
99	24.08	318.2999	[M + H]^+^	Phytosphingosine	554-62-1	C_18_H_39_NO_3_	II	82	E, M; #4, #5
100º	24.10	882.2875	[M + NH4]^+^	Cyclic 12LA	-	C_36_H_48_O_24_	I	-	E; #6
101º	24.32	676.2963	[M + NH4]^+^	Linear 2TPA-1AA-3BD	-	C_34_H_42_O_13_	I	-	E; #6
102º	24.33	856.4323	[M + NH4]^+^	Linear 1TPA-3AA-4BD	-	C_42_H_62_O_17_	I	-	E; #6
103º	24.41	954.3080	[M + NH4]^+^	Cyclic 13LA	-	C_39_H_52_O_26_	I	-	E; #6
104º	24.51	928.4896	[M + NH4]^+^	Linear 1TPA-3AA-5BD	-	C_46_H_70_O_18_	I	-	E; #6
105º	24.65	748.3532	[M + NH4]^+^	Linear 2TPA-1AA-4BD	-	C_38_H_50_O_14_	I	-	E; #6
106º	24.83	984.4794	[M + NH4]^+^	Linear 1TPA-4AA-4BD	-	C_48_H_70_O_20_	I	-	E; #6
107º	25.07	638.3167	[M + NH4]^+^	Cyclic 1TPA-2AA-3BD	-	C_32_H_44_O_12_	III	-	E, M; #6
108º	25.18	876.4003	[M + NH4]^+^	Linear 2TPA-2AA-4BD	-	C_44_H_58_O_17_	I	-	E; #6
109º	25.28	948.4576	[M + NH4]^+^	Linear 2TPA-2AA-5BD	-	C_48_H_66_O_18_	I	-	E; #6
110	25.74	403.2340	[M + H]^+^	Tributyl acetylcitrate (ATBC)	77-90-7	C_20_H_34_O_8_	60	89	E, M; #6
111º	26.33	658.2849	[M + NH4]^+^	Cyclic 2TPA-1AA-3BD	-	C_34_H_40_O_12_	III	-	E, M; #6
112	26.52	343.2117	[M + H]^+^	Tributyl aconitate	7568-58-3	C_18_H_30_O_6_	I	-	E, M; #6
113º	26.79	858.3901	[M + NH4]^+^	Cyclic 2TPA-2AA-4BD	-	C_44_H_56_O_16_	III	-	E, M; #6
114	27.27	496.3391	[M + H]^+^	1-Palmitoyl-sn-glycero-3-phosphocholine	17364-16-8	C_24_H_50_NO_7_P	III	88	E; #2, #3, #5
115	29.60	371.3158	[M + H]^+^	Bis(2-ethylhexyl) adipate	103-23-1	C_22_H_42_O_4_	18	93	E, M; #1, #2, #3, #4, #5, #6

Note: RT standards for retention time. * in the Nº column represent melamine derivatives with their chemical name generated by Chemdraw, while º stands for PLA and PBAT oligomers and their derivatives, with their chemical name expressed as the type of the molecule, either cyclic or linear, plus the number of each monomer; LA = lactic acid; AA = adipic acid; BD = 1,4-buanediol; TPA = terephthalic acid; EtOH = ethanol; MeOH = methanol; in the SML or CR column, if a compound has specific migration limit (SML) in the EU 10/2011 regulation, then the value is the SML in mg/kg unit, otherwise Cramer rules classification (Cramer I, II, and III) predicted by Toxtree is given. The Score is the measure of spectra similarity between the acquired spectrum and the reference spectrum given by MS-DIAL. If a compound was identified by library matching, then the score given by MS-DIAL was taken. Otherwise, “-” was used. In the Sample column, letters before the semi column represent in which extracts the compounds were detected, where E standards for ethanol extract, M standards for isooctane extract (redissolved in methanol), and H stands for 4% acetic acid extract. # in the Sample column is a symbol of sample code.

## Data Availability

The datasets generated during and/or analyzed during the current study are available from the corresponding author upon reasonable request.
